# Structure of the transmembrane domain of human nicastrin-a component of γ-secretase

**DOI:** 10.1038/srep19522

**Published:** 2016-01-18

**Authors:** Yan Li, Lynette Sin Yee Liew, Qingxin Li, CongBao Kang

**Affiliations:** 1 Experimental Therapeutics Centre, Agency for Science, Technology and Research (A^*^STAR), Singapore, 138669 Singapore; 2 Institute of Chemical & Engineering Sciences, Agency for Science, Technology and Research (A^*^STAR), Singapore, 138669 Singapore

## Abstract

Nicastrin is the largest component of γ-secretase that is an intramembrane protease important in the development of Alzheimer’s disease. Nicastrin contains a large extracellular domain, a single transmembrane (TM) domain, and a short C-terminus. Its TM domain is important for the γ-secretase complex formation. Here we report nuclear magnetic resonance (NMR) studies of the TM and C-terminal regions of human nicastrin in both sodium dodecyl sulfate (SDS) and dodecylphosphocholine (DPC) micelles. Structural study and dynamic analysis reveal that the TM domain is largely helical and stable under both SDS and DPC micelles with its N-terminal region undergoing intermediate time scale motion. The TM helix contains a hydrophilic patch that is important for TM-TM interactions. The short C-terminus is not structured in solution and a region formed by residues V697-A702 interacts with the membrane, suggesting that these residues may play a role in the γ-secretase complex formation. Our study provides structural insight into the function of the nicastrin TM domain and the C-terminus in γ-secretase complex.

Mature γ-secretase is a four-component intramembrane protease and cleaves transmembrane (TM) domains of single-span membrane proteins^1^. The γ-secretase is important for development of Alzheimer’s disease that is characterized by formation of β-amyloid plaque in the patient brain^2^. Amyloid precursor protein is first cleaved by β-secretase to produce a 99-residue transmembrane fragment (C99)^3^,^4^. C99 is further cleaved by γ-secretase to produce a series of amyloid peptides (Aβ) among which the Aβ42 is prone to aggregation to form β-amyloid plaque-a step in the development of Alzheimer’s disease^5^.

The γ-secretase complex is composed of presenilin, presenilin enhancer 2 (Pen2), nicastrin, and anterior pharynx defective 1 (Aph-1)^6^. The subunit assembly of γ-secretase TM regions has been revealed by recent structural studies^7^,^8^,^9^,^10^. The TM helices of γ-secretase forms a horseshoe-shaped structure^10^. Presenilin is the catalytic subunit and contains 9 TM domains. Presenilin becomes an N-terminal fragment (NTF) formed by TMs 1–6 and a C-terminal fragment (CTF) formed by TMs 7–9 through a protease cleavage during secretase complex assemble^11^. The two aspartic acid residues in TM6 and TM7 are located on the convex side of TM regions, which may make it easy for the substrate protein to access^7^. Aph-1 is a seven-TM membrane protein and its function is for γ-secretase complex assembly^12^,^13^. Pen2 is the smallest component and it is thought to be essential for presenilin maturation and the γ-secretase protease activity^12^,^14^. Pen2 was shown to have 3 TMs instead of 2 TMs with only one TM helix coming across the cell membrane^7^,^15^. TM1 and TM3 of Pen2 are packed closely with TM3 of presenilin^7^.

Nicastrin is a 709-residue, type-I membrane protein and is the largest component of the γ-secretase complex^16^. It contains a large extracellular domain with multiple glycosylation sites, one TM domain, and a very short C-terminal tail that may not be necessary for γ-secretase assembly^9^,^17^. Studies have demonstrated that the extracellular domain is important for substrate recruitment^16^,^18^,^19^ and the N-terminal juxtamembrane region is essential for complex formation^20^. Structural studies revealed that the extracellular domain contains two lobes and is located above the TM regions of the secretase interacting with the extracellular loops of the TMs of other subunits^7^. Nicastrin TM interacts with TMs of Aph-1^7^. Although the packing of the 20 TMs of γ-secretase has been resolved, it is still useful to have detailed structural information for the TM regions of individual subunits to understand their function in γ-secretase complex. As nicastrin contains a single TM and a short C-terminus, we investigated its structure using solution NMR spectroscopy to understand its structure and dynamic in solution.

In this study, we expressed and purified a construct containing residues A664-Y709 of human nicastrin. This construct includes the TM domain and the short C-terminal region of human nicastrin. We used NMR spectroscopy to characterize its structure in both sodium dodecyl sulfate (SDS) and dodecylphosphocholine (DPC) micelles. The TM domain of nicastrin forms a helix in detergent micelles and the N-terminal part of TM domain may undergo conformational exchanges. The C-terminus is flexible while several residues interact with membrane. The current study provides additional information to understand the structure and function of the TM region and the C-terminus of the human nicastrin.

## Results

### Secondary structure of nicastrin TM region in both SDS and DPC micelles

**Figure 1:**
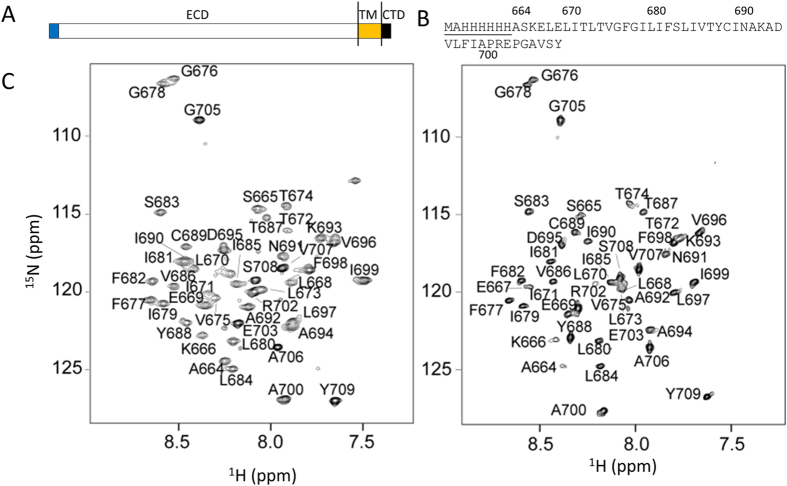
Assignment of nicastrin TM domain and C-terminus. (**A**) Domain organization of human nicastrin. The N-terminal signal peptide, extracellular domain, TM domain, and C-terminus are shown in blue, white, brown, and black, respectively. (**B**) sequence of the nicastrin construct used in this study. Underlined residues are artificial residues to aid in protein purification. (**C**) Assigned ^1^H-^15^N-HSQC spectra of nicastrin in SDS micelles (left panel) and in DPC micelles (right panel). The cross peaks are labeled with residue name and residue number.

**Figure 2:**
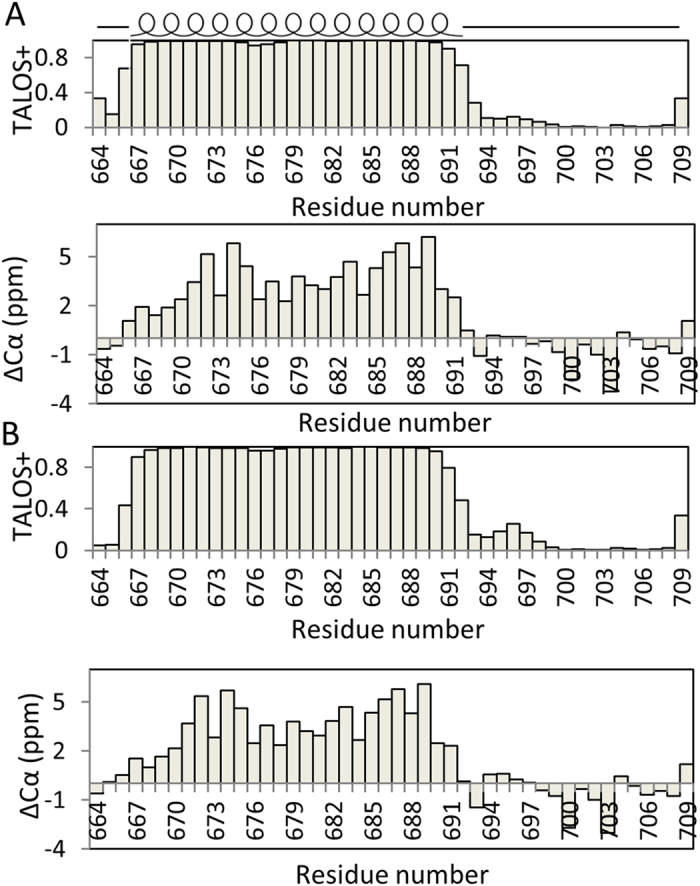
Secondary structure of nicastrin in SDS (**A**) and DPC micelles (**B**). Upper and lower panels are secondary structural prediction based on TALOSN and Cα chemical shift, respectively. ΔCα is the difference between chemical shift of a residue and its random coil value.

An N-terminal histidine-tagged construct of human nicastrin (residues 664 to 709) containing the TM domain and C-terminus of human nicastrin was made for structural studies (Fig. 1). This construct can be expressed in *E. coli* in enough quantity for structural studies (Figure S1). Recombinant protein was first expressed into inclusion bodies that were dissolved in a urea solution, followed by addition of Ni^2+^-metal ion affinity resin and purification into SDS and DPC micelles^21^,^22^. Purified nicastrin exhibited well-resolved NMR spectra in both SDS and DPC micelles (Fig. 1). The dispersion of the cross peaks in the ^1^H-^15^N-HSQC spectrum is within 7.0 to 9.0 ppm for the ^1^H chemical shift. Such narrow chemical shifts dispersion was observed for folded helical membrane proteins^23^, suggesting that purified protein adopted helical structures under such conditions. Backbone assignments of nicastrin in both SDS and DPC micelles were achieved using conventional 3D experiments and have been deposited in BioMagResBank (BMRB entry 25817 and 25818). Complete backbone resonance assignments for the sample in SDS micelles were accomplished (Fig. 1B). Nearly complete backbone assignments for nicastrin in DPC micelles were obtained except for residues including E670, L671, L673 and V675 exhibited line broadening in the ^1^H-^15^N-HSQC spectrum (Fig. 1C). Broadened peaks were also observed for these residues when nicastrin was reconstituted in SDS micelles. The peak intensity of the cross peaks in the ^1^H-^15^N-HSQC spectrum is very sensitive to the environment. Broadened peaks can be caused by its interaction with detergent or existence of multiple conformations. This result indicates that these residues may be different from other residues in the TM region and they may have exchanges with the environment. The secondary structure was determined using both chemical shift index based on the Cα chemical shifts and TALOSN analysis of the assigned chemical shifts (Fig. 2). The results demonstrate that the nicastrin contains one helix formed by residues E667 to I691. Both N- and C-termini are not structured (Fig. 1). NOE connectivity also showed the presence of the helical structure in the construct (Figure S3).

### Structure of nicastrin in SDS and DPC micelles

**Figure 3:**
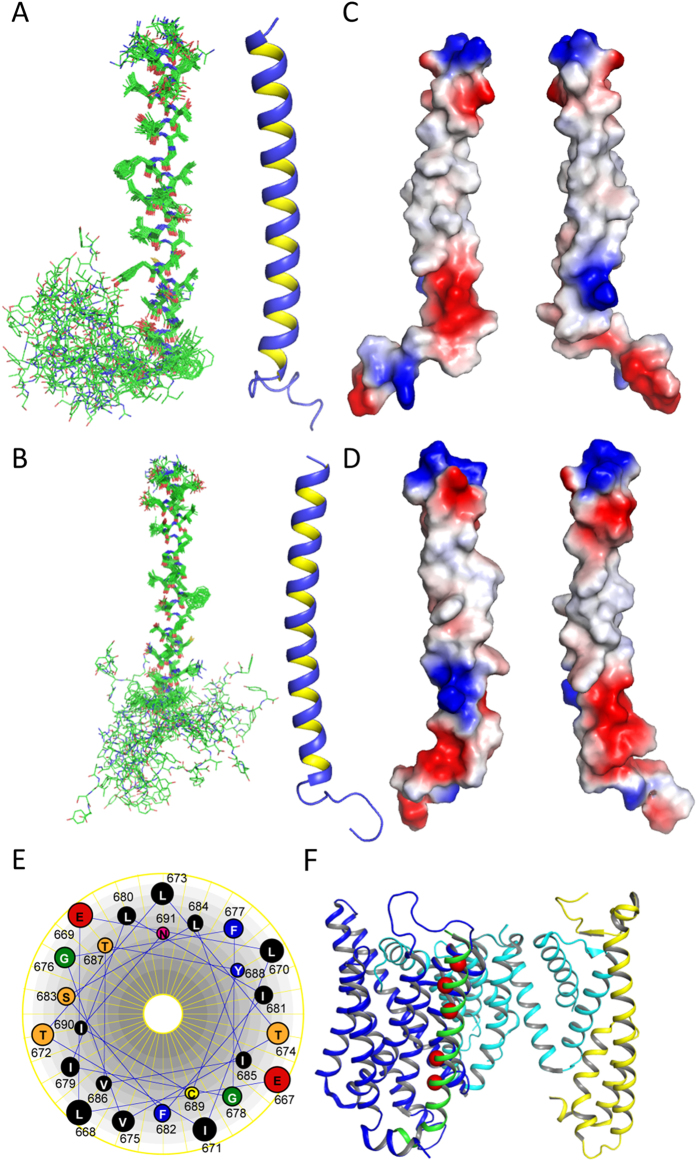
Structure of the nicastrin TM domain the its C-terminus. Structures of nicastrin in SDS (**A**) and DPC (**B**) micelles. Left panel is the ensemble of 20 superimposed structures that are aligned based on the helix. Right panels are the cartoon structure of nicastrin. C, D, Color-coded electrostatic surface potential for the TM helix of human nicastrin in SDS (**C**) and DPC (**D**) micelles. The figure was generated using PyMOL (www.pymol.org). The positive, hydrophobic and negative surfaces are shown in blue, white and red, respectively. E, Helix wheel plot of the helix of nicastrin TM domain. F. Structure of γ-secretase TM helices obtained from Cryo-EM. The Cryo-EM structure of γ-secretase (PDB 5a63) is shown. The TM domains of presenilin, Pen2, Aph-1 and nicastrin are shown in cyan, yellow, blue, and green, respectively. The extracellular domain of nicastrin is not shown for clarity. The residues from nicastrin TM domain forming a hydrophilic patch are shown in red sphere.

Solution structures of nicastrin in both SDS and DPC micelles were determined using XPLOR-NIH^24^ based on NMR restraints including dihedral angles and side chain angles derived from TALOSN^25^, NOEs derived from a NOESY experiment and hydrogen bond restraints obtained from an H-D exchange experiment (Figure S2 and Table S1). Structures have been deposited in the protein Data Bank under accession codes 2N7Q and 2N7R, respectively. There was no significant structural difference observed for nicastrin in both SDS and DPC micelles. Nicastrin contains an α-helix encompassing residues E667-V696 (Fig. 3). The well defined region contains residues E667 to I691 because the NOEs were observed (Figure S3, Fig. 3).The C-terminus of nicastrin is not structured in solution. Surface-charge representation showed that the middle of the TM region is mainly hydrophobic, which is not surprising for a TM domain. Both C-and N-terminal regions of the TM domain contain charged surfaces that may be important for TM-TM or membrane interactions. Sequence analysis revealed that there is no GXXXG (X, any residue) or polar-XX-polar (polar, polar residues) motif that are important for TM-TM interactions^26^. It contains three G-XXX-G like motifs including T_672_XXXG_676_, T_674_XXXG_678_, and S_683_XXXT_687_ and these motifs may be important for its function such as involving in protein-protein interactions. Helix view analysis showed that shows that there is a hydrophilic patch formed by residues E669, T672, G676, S683, and T687 (Fig. 3E). Recent high resolution cryo-EM structure suggested that these residues are in the interface between the TM domain of nicastrin and Aph-1 (Fig. 3E)^27^, confirming their importance in TM-TM interactions in the γ-secretase complex. There is a possible hydrophilic patch formed by residues T674, E667, and G678 on the opposite side of the Aph-1 binding side. These residues may not be stable or undergo exchanges in hydrophobic environment such as detergent micelles, which may explain the line broadening of their nearby residues. These residues may have a similar function to that of the residues interacting with the TM domains of Aph-1. Due to their location to the substrate entry site, these residues may have an interaction with substrate such as C99 that also contains hydrophilic residues in its TM domain^28^.

### Dynamic analysis of nicastrin in micelles

**Figure 4:**
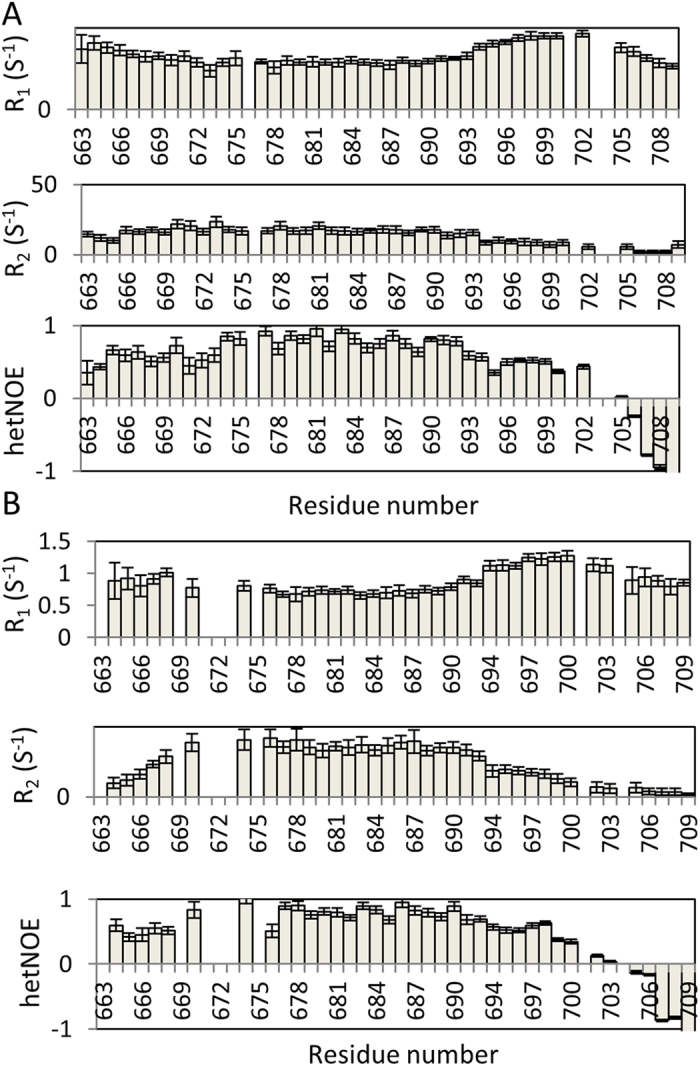
^15^N-R_1_, R_2_ and hetNOE measurements of nicastrin TM domain and the C-terminus in SDS (**A**) and DPC (**B**) micelles. The data were collected on a Bruker Avance II 700 MHz magnet at 313 K.

To understand the dynamics of nicastrin TM domain and C-terminus in micelles, spin-lattice relaxation rate constants (R_1_), spin-spin relaxation rate constants (R_2_), and heteronuclear NOE (hetNOE) relaxation data were collected (Fig. 4). In consistent with the TALOSN prediction, the N-terminal region (664–669) of the construct is flexible in both micelles, characterized by the low hetNOE values. Residues from 674 to 690 were shown to be stable in both SDS and DPC micelles, characterized by the high hetNOE/R_2_ and low R_1_ values, which is not surprising because this region is the TM region (Fig. 4). Residues showed broadened peaks in DPC micelles exhibited slightly lower hetNOE (>0.5) values than those in the rigid region (close to 0.8). Some residues such as T679 exhibited a slightly higher R_2_ value than those of other TM residues, suggesting that this residue and its nearby residues may undergo intermediate exchanges with the environment. Similar result was also observed in other TM protein^23^. The C-terminal region of nicastrin contains two regions. One region contains residues 694-700 and the other one contains residues 702-709. The later region is highly disordered evidenced by the low and negative hetNOEs (Fig. 4). The region close to the TM region is less stable than the TM helix, which may arise from the fact that it is not a transmembrane region.

### Topology of nicastrin TM domain and its C-terminus

**Figure 5:**
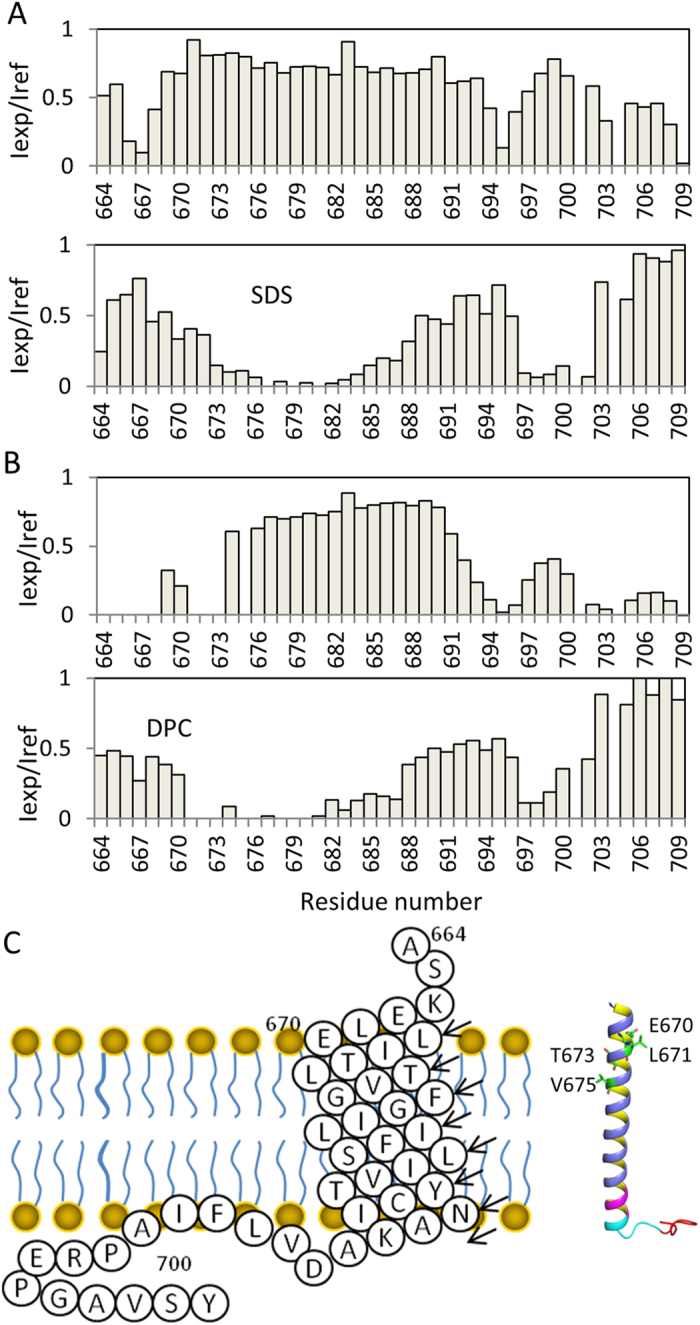
Topology of nicastrin TM domain and the C-terminus in SDS (**A**) and DPC (**B**) micelles. The peak intensities of residues in the absence (Iref) and presence of 2 mM 16-DSA (lower panel) or 4 mM gadolinium (upper panel) (Iexp) were plotted against residue number. (**C**) Diagram of nicastrinTM domain and the C-terminus in membrane. Right panel is one of the structures determined in SDS micelles. Residues having broadened peaks are shown in sticks and green. C-terminal residues are less stable than those in TM domain are shown in purple. Residues interacting with membranes are shown in cyan and C-terminal flexible residues are shown in red.

We next examined the topology of the nicastrin in solution using a water soluble paramagnetic probe, gadolinium and a lipid soluble probe, 16-DOXYL-steric acid (16-DSA). The paramagnet-induced intensity changes in the ^1^H-^15^N-HSQC spectra were analyzed and plotted (Fig. 5). Compared with nicastrin in DPC micelles, few residues were solvent-exposed when nicastrin was reconstituted in SDS micelles (Fig. 5). This may arise from the fact that SDS is a charged detergent that may interact with negatively charged residues, which makes them protect from solvent exposure. Despite this difference, the TM domain of nicastrin was protected from gadolinium and broadened by addition of 16-DSA in both SDS and DPC micelles, demonstrating the residues L670 to I690 form the TM domain of nicastrin. H-D exchange experiment also showed that residues from the TM helix were protected from exchanges (Fig. 5 and Figure S2). The N-terminal region and the C-terminus containing residues 702 to 706 are exposed to the solvent (Fig. 5). A sequence containing residues V696-A700 from the C-terminal juxtamembrane region is lipid accessible (Fig. 5). This five-residue sequence contains hydrophobic residues. Its interaction with micelles suggests that this region may interact with cell membrane or interact with a hydrophobic region of other proteins, which may be important for γ -secretase complex function.

## Discussion

High resolution structures of the extracellular domain of nicastrin and the γ -secretase complex shed light on the assembly of the important enzyme, providing valuable information to understand the structure and function of γ -secretase complex^7^,^9^,^10^. The folding of the γ -secretase TM domains has been well presented^7^. In current study, we solved the structure of the TM domain and the C-terminus of human nicastrin in both SDS and DPC micelles using NMR spectroscopy. We show that the nicastrin TM domain is helical in both SDS and DPC micelles. The TM helix of nicastrin obtained in current study is similar to the structural model revealed by cryo-EM study (Fig. 3). The length of the helix in SDS and DPC micelles is compatible with the one from the Cryo-EM structure (Fig. 3). There is a cysteine residue and G-XXX-G like motif present in the TM region of nicastrin (Fig. 1). It is well known that the domain interact with Aph-1 while it is still unclear whether this region can form dimers. Although our current studies suggested that the protein is monomeric in both SDS and DPC micelles based on the HSQC spectrum and relaxation analysis (Figs 1 and 4), we found that higher concentration of SDS (2%) needs to be present in the urea buffer during samples preparation in order to obtain a sample with dispersed cross peaks in the ^1^H-^15^N-HSQC spectrum (data not shown). This suggests that the TM domain may have a tendency to form dimers or oligomers.

We also provide additional information to understand the structure of nicastrin in current study. Firstly, we showed that the TM domain formed similar structures in both SDS and DPC micelles (Fig. 3). This result suggests that the TM domain is stable and may not undergo significant conformational changes when the membrane condition is altered. Secondly, we provide structural information for the C-terminus. Nicastrin contains a short C-terminus and this region was shown not to be important for γ-secretase assembly^9^,^17^. We provide structural information for the C-terminus. Our study clearly indicated that the residues formed by V696-A700 were solvent protected and may interact with micelles/membranes (Fig. 5). These residues might be important for interaction with cell membrane or with the other subunits of γ-secretase. Indeed, the recent structure of the γ -secretase showed that this region interacts with Aph-1^27^. These membrane-interacting residues are important for Aph-1 interaction, which may stabilize the complex structure. Thirdly, we provide dynamic analysis for both TM domain and the C-terminus of human nicastrin. The TM domain is stable under micelle conditions and the C-terminus is highly dynamic. The C-terminal Aph-1 interacting residues may change their conformation under certain conditions, suggesting that they may play a role in complex stabilization or assembly. Lastly, NMR is a very useful tool to study protein structure and conformational changes. The intensity of the cross peaks in the ^1^H-^15^N-HSQC spectrum is very sensitive to the environment. Residue exhibiting a broadened peak in the spectrum may arise from its interaction with a ligand or existence of multiple conformations. We found that residues such as E670, L671, E673 and V675 exhibited broadened peaks in the spectrum, suggesting that they may undergo conformational exchanges or have different characteristics from other residues in the TM region.

In this study, the structure of the TM domain and the C-terminus of human nicastrin is solved in detergent micelles. There is a debate that whether detergent micelles can mimic the physiological conditions. Indeed, SDS micelles are widely used in denature of water soluble proteins. Accumulated NMR studies have shown that detergent micelles can serve as a membrane-mimicking system for structural and functional studies of membrane proteins using solution NMR spectroscopy^29^,^30^. An NMR study on the CTF of presenilin was carried out in SDS micelles. Although the CTF structure in SDS micelles is different from that observed in the cryo-EM structure, the difference observed in different studies may suggest that other γ-secretase subunits are important for the folding of presenilin. The dimeric structure of the TM domain of glycophorin A was solved in SDS micelles^31^ in which the TM domain forms stable dimer structures, suggesting that SDS micelles can be a useful system for membrane protein structures. DPC is a neutral detergent and different from SDS that is a charged detergent. It is the most commonly used detergent in structural determination of membrane proteins due to its close structure to the cell membrane^32^. Several membrane proteins including multi-span membrane proteins have been shown to be folded and functional in DPC micelles^33^,^34^. For the nicastrin, we observed that its folding in SDS and DPC micelles is close to the physiological conditions because these two detergents have been proven to be a suitable system for NMR study of membrane proteins with small or medium size^31^,^35^ (Fig. 3). The structures in these micelles are similar to the one obtained from cryo-EM studies^7^. Difference was also observed when nicastrin was reconstituted in DPC and SDS micelles. Line broaden of cross peaks of several residues at the N-terminal part of the helix were observed in DPC micelles. These residues are localized at the opposite site of the binding interface between nicastrin and Aph-1, suggesting that the stability of these residues is sensitive to the environment. These residues may have interchanges, indicating that they may have some functions such as interacting with the substrate as aforementioned. Although detergent micelles are good systems for membrane protein structural study, it has been noted that they may not be a good system for some membrane proteins because the curvature of micelles may have effect on protein folding^36^. A recent study showed that the curvature of the micelle surface may dramatically affect the juxtamembrane structure of human Respiratory Syncytial virus small hydrophobic protein^37^. Further study in lipid systems such as isotropic bicelles will provide more information to understand the role of the TM domain in secretase complex and TM-TM interactions. It has been also found that the composition of the lipid system used in NMR studies can affect structure such as helix tilt and dynamic of the TM domain^38^,^39^. Further NMR studies of the transmembrane helices of γ-secretase subunits in lipid bilayer system will provide more information to understand the structure and dynamics of the enzyme complex. Due to the large size of the protein/lipid complex, solid NMR spectroscopy will be suitable for such studies^40^. Accumulated studies have shown that solid-state NMR experiments provided additional information to understand the structure and functional of membrane proteins containing both single- and multi-span transmembrane helices^29^,^30^,^41^,^42^,^43^. The cause of Alzheimer’s disease is a complicated step and involves in several proteins. The aggregation of amyloid β peptides has been thoroughly studied using NMR spectroscopy, which provide insight into the mechanism of the disease^40^,^44^. The structural model of the TM helices of the secretase has been obtained using Cryo-EM^7^,^27^. Our study showed the possibility to study the structure of the TM domain of nicastrin. Further NMR study on the secretase complex will provide more insight into the dynamic of this enzyme complex, which will be helpful to understand the mechanism of Alzheimer’s disease.

In summary, we present the solution structure of the TM domain and the short C-terminus of nicastrin. Structural study revealed that there is a helix present in TM domain. There is a region at the C-terminal JM region of nicastrin was shown to be solvent protected, which may be important for its function. Our study provides a basis to understand the structure of the TM domain of nicastrin, which will be useful to understand its role in γ-secretase assembly and substrate binding.

## Methods

### Sample preparation

The cDNA for encoding residues A664-Y709 of human nicastrin (www.uniprot.org access id Q92542) was synthesized (Genscript). The cDNA was amplified and cloned into the NdeI and XhoI sites of pET29b to generate a plasmid that encodes a protein sequence including the TM domain and the C-terminal region of nicastrin with eight residues (MAHHHHHH) at its N-terminus. The resulting plasmid was transformed in *Escherichia coli* (*E. coli*) (DE3) competent cells and plated onto LB plates supplied with 30 μg/ml kanamycin. Two to three colonies were picked up from the plate and inoculated a 20 ml start culture at 37 °C overnight. The overnight culture was then inoculated into 1 l culture of M9 medium. When OD_600_ of the culture reached 0.8–1.0, nicastrin protein was induced for 12 h at 37 °C by adding β-D-1-thiogalactopyranoside to 1 mM. Isotope-labelled proteins were also induced using the similar method except using ^13^C-glucose and ^15^NH_4_Cl as carbon and nitrogen sources^45^. The *E. coli* cells were obtained by centrifugation at 10,000 ×g for 10 min at 4 °C, and the cell pellets were re-suspended into a lysis buffer that contained 50 mM Tris-HCl, pH 7.8, 300 mM NaCl, and 2 mM β-mercaptoethanol and were then broken up by sonication in an ice bath. Inclusion bodies were harvested by centrifugation at 20,000 × g for 20 min. Inclusion bodies were then solubilized in a urea buffer that contained 8 M urea, 300 mM NaCl, 70 mM SDS, 20 mM Tris-HCl, pH7.8^46^,^47^. Protein was purified using Ni^2+^-NTA resin in a gravity column. Imidazole was removed using a PD column and the final NMR sample contains 0.8 mM nicastrin, 20 mM sodium phosphate, pH6.5, 200 mM DPC (100 mM SDS) and 1 mM DTT.

### Nicastrin backbone assignment

All the NMR spectra were collected at 313 K (40 °C) on a 600 or 700 MHz Bruker Avance spectrometer equipped with a cryoprobe. Uniformly ^13^C/^15^N-lableled nicastrin was used for backbone data collection. Backbone resonance assignment was obtained based on two- (2D) and three-dimensional (3D) experiments including 2D-HSQC, 3D-HNCACB, 3D-HNCOCACB, 3D-HNCOCA, 3D-HNCA, and 3D-HNCO. 3D-HBHACONH and NOESY-TROSY (120 ms mixing time) experiments were collected for proton chemical shift assignment. All the pulse sequences were from standard Bruker pulse program library (Topspin 2.1). Spectra were processed with NMRPipe^48^ or Topspin and analyzed using NMRView^49^ and CARA (http://www.mol.biol.ethz.ch/groups/wuthrich_group).

### Nicastrin relaxation experiment

*R*_1_, *R*_2_ and ^1^H-^15^N heteronuclear NOE (hetNOE) experiments^50^ were measured at 313 K using a ^15^N-labeled nicastrin on a Bruker Avance II 700 MHz spectrometer. For *T*_1_ measurement, the relaxation delays of 50, 80, 130, 330, 470, 630, 800, 900, 1000, 1200, 1400, 1600 and 1800 ms were recorded. For *T*_2_ measurement, the data were acquired with delays of 17, 34, 51, 68, 85, 102, 119, 136, and 153 ms. The hetNOE values were obtained using two datasets that were collected with and without initial proton saturation for a period of 3 s^51^.

### Paramagnetic probe accessibility NMR measurement

To probe residues that are exposed to the solvent, accessibility of residues to water-soluble probes such as gadolinium and lipid soluble probe 16-DSA, was evaluated using ^1^H-^15^N-HSQC experiments^52^. Uniformly ^15^N-labeled nicastrin was prepared in a buffer that contained 20 mM sodium phosphate, pH6.5, 200 mM DPC (100 mM SDS) and 1 mM DTT with 0.8 mM nicastrin. A gadolinium solution containing 50 mM GdCl_3_ and 150 mM ethylenediaminetetraacetic acid (EDTA) was freshly prepared before the experiment. ^1^H-^15^N-HSQC spectra of nicastrin in the absence and presence of 4 mM gadolinium were acquired and analyzed^53^. For the 16-DSA experiment, 16-DSA was first dissolved in D-methanol to 30 mM concentration. Aliquots of 16-DSA were prepared and methanol was then air-dried. ^1^H-^15^N-HSQC spectra of nicastrin in the absence and presence 2 mM 16-DSA were recorded and processed.

### Structure determination

The backbone dihedral angle restraints were generated using TALOSN based on the assigned chemical shifts^54^. The peak intensities of the cross peaks from the NOESY spectra were converted to distance restraints. Hydrogen bond restraints were obtained from H-D exchange experiment^55^. To prepare for an H-D exchange experiment, a ^15^N-labled nicastrin in SDS or DPC was first prepared as aforementioned. Sample was then frozen in liquid nitrogen. The sample was lyophilized before D_2_O was added to the lyophilized sample for data acquisition. The spectra before and after lyophilization were also compared to make sure that there is no change during the frozen and lyophilization steps. The residues that protected from exchanges are considered to form a hydrogen bond with other residues. The upper and lower distances used in hydrogen bond restraints were set to 2.8 and 1.8 Å, respectively. Structure determination was carried out using XPLORE-NIH with python interface^24^,^56^,^57^. Simulated annealing from a randomized template was performed and energy minimization were carried out as previously described^22^,^47^. Protein structure was analyzed by MOLMOL^58^, PyMOL (www.pymol.org) and PROCHECK-NMR^59^.

## Additional Information

**Accession Codes**: Coordinates of structures in SDS and DPC micelles have been deposited in the protein Data Bank under accession codes 2N7Q and 2N7R, respectively. Assignments of nicastrin in SDS and DPC micelles have been deposited in the Biological Magnetic Resonance Bank (BMRB) under accession numbers 25817 and 25818, respectively.

**How to cite this article**: Li, Y. *et al*. Structure of the transmembrane domain of human nicastrin-a component of γ-secretase. *Sci. Rep*. **6**, 19522; doi: 10.1038/srep19522 (2016).

## Supplementary Material

Supplementary Information
