# Supplementary material for: Structure of the transmembrane domain of human nicastrin-a component of γ-secretase
Source: Sci Rep. 2016 Jan 18;6:19522. doi: 10.1038/srep19522 (PMC4726005; doi:10.1038/srep19522)
Supplement: Supplementary Information [file srep19522-s1.pdf]

Supplementary Information

Structure of the transmembrane domain of human nicastrin-a component of γ-secretase

Yan Li[1], Lynette Sin Yee Liew[1], Qingxin Li[2] & CongBao Kang[1]

[1]Experimental Therapeutics Centre, [2]Institute of Chemical & Engineering Sciences, Agency for Science, Technology and Research (A*STAR), Singapore, 138669 Singapore

To whom correspondence should be addressed: CongBao Kang, 31 Biopolis Way Nanos, #03-01,01Singapore. Tel: 65-64070602; Fax: 65-64788768; Email: cbkang@etc.a-star.edu.sg. Qingxin Li, email: li_qingxin@ices.a-star.edu.sg

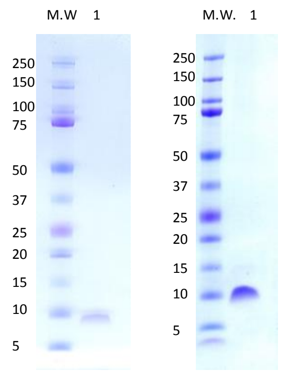

Figure S1 Purification of nicastrin TM domain and the C-terminus from *E. coli*. Purification of human nicastrin in SDS (left panel) and DPC micelles (right panel). M.W. is molecular weight. 1 is the protein sample.

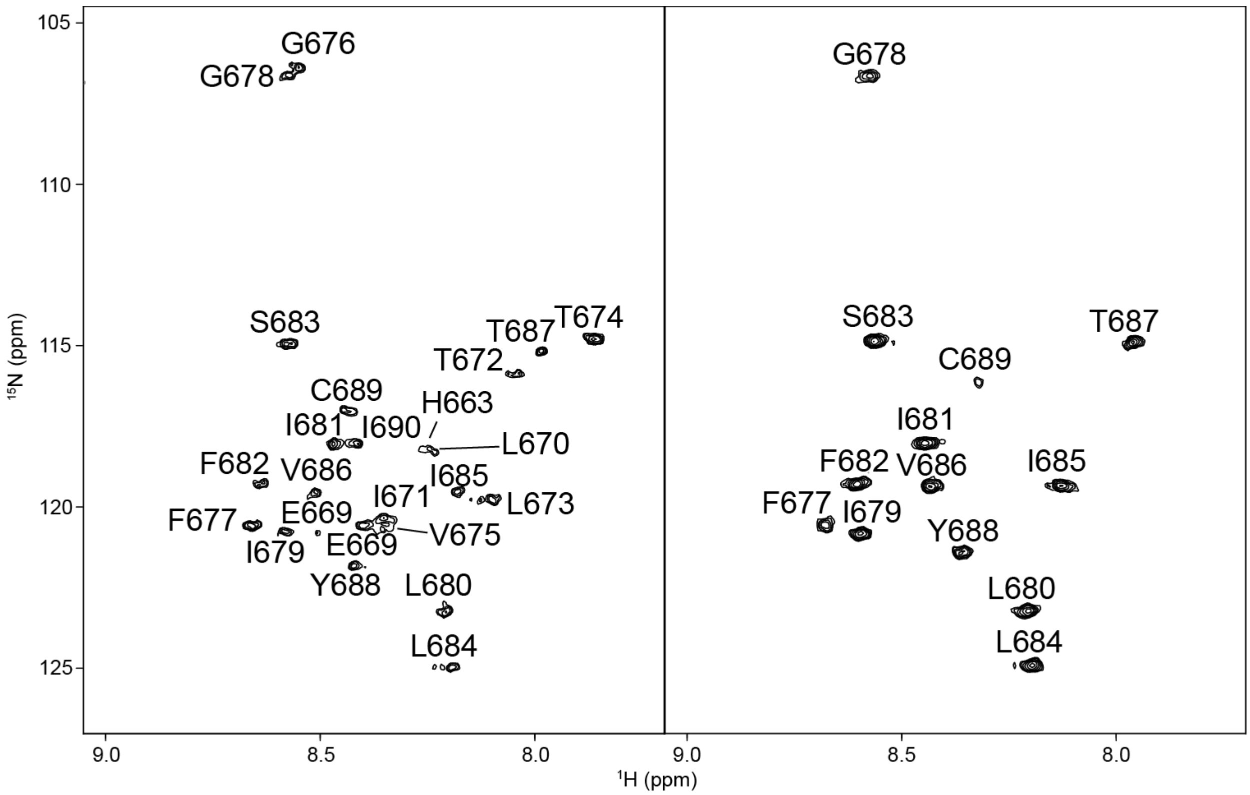

Figure S2 H-D exchange experiment. Left and right panels are the ^{1}H-^{15}N-HSQC spectra of ^{15}N-labeled nicastrin in SDS and DPC micelles, respectively.

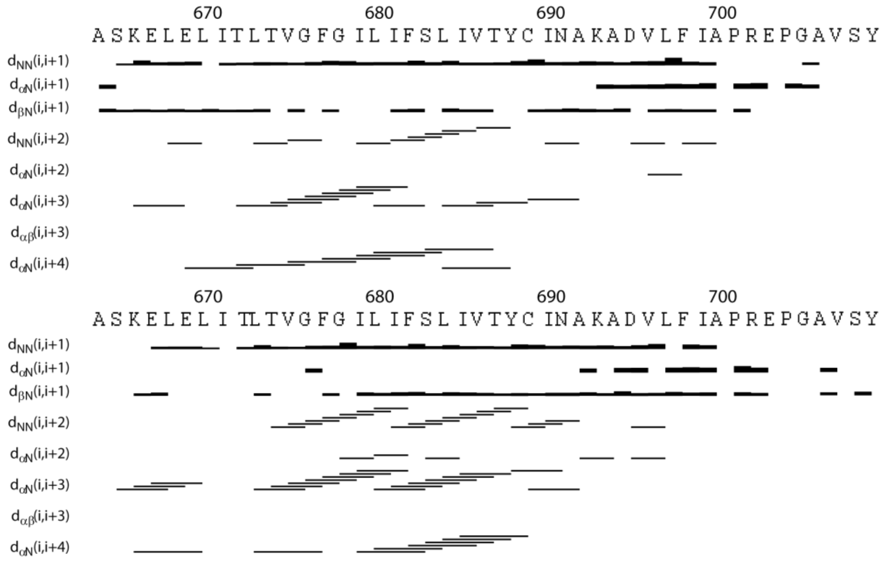

Figure S3 NOE connectivity of nicastrin in SDS (upper panel) and DPC micelles. This figure is generated using CYANA [1] based on obtained NOEs.

Table S1 Summary of the 20 structures of nicastrin in SDS and DPC micelles

	SDS	DPC
**Number of unambiguous NOEs**	216	258
Short range (|i–j|≤ 1)	183	206
Medium-range (1 <|i–j|< 5)	33	52
Long-range (|*i*–*j*| > 4)	0	2
Number of angle constraints[a]	98	100
Number of hydrogen-bond restraints	22	13
**Number of restraint violations[b]**		
Total number of restraint violations > 0.5 Å	0	0
Total number of dihedral angle constraints > 5°	0	0
**Ramachandran plot statistics[c] (%)**		
Residues in most favored regions	82.1	89.7
Residues in additionally allowed regions	15.4	10.3
Residues in generously allowed regions	2.6	0
Residues in disallowed regions	0	0
**Average RMSD to mean (Å)**		
Backbone (residues 666-699)	0.53 ± 0.19 Å	0.62 ± 0.24 Å
Heavy atoms (residues 666-699)	1.10 ± 0.20 Å	1.20 ± 0.23 Å

[a] the angle restraints also include chi-1 angles that were obtained from TALOSN[2].

[b]There are no distance violations greater than 0.5 Å or dihedral angle violations greater than 5°.

[c] The Ramachandran plot was obtained using PROCHECK-NMR based on the conformer with lowest energy. The analysis was conducted for residues from A664 to Y709.

References

1. Guntert P. Automated NMR structure calculation with CYANA. *Methods Mol Biol* **278**, 353-378 (2004).

2. Shen Y, Bax A. Protein structural information derived from NMR chemical shift with the neural network program TALOS-N. *Methods Mol Biol* **1260**, 17-32 (2015).
